# The Function of Tranexamic Acid to Prevent Hematoma Expansion After Intracerebral Hemorrhage: A Systematic Review and Meta-Analysis From Randomized Controlled Trials

**DOI:** 10.3389/fneur.2021.710568

**Published:** 2021-09-24

**Authors:** Zeya Yan, Shujun Chen, Tao Xue, Xin Wu, Zhaoming Song, Zongqi Wang, Zhouqing Chen, Zhong Wang

**Affiliations:** ^1^Department of Neurosurgery & Brain and Nerve Research Laboratory, The First Affiliated Hospital of Soochow University, Suzhou, China; ^2^Department of Neurology, The First Affiliated Hospital of Soochow University, Suzhou, China

**Keywords:** spontaneous intracerebral hemorrhage, tranexamic acid, meta-analysis, randomized controlled trials, hematoma, modified Rankin Scale

## Abstract

**Objectives:** The clinical results caused by spontaneous intracerebral hemorrhage (ICH) are disastrous to most patient. As tranexamic acid (TXA) has been proved to decrease the influence of ICH, we conducted this research to explore the function of TXA for the prognosis of ICH compared with placebo.

**Methods:** We searched MEDLINE, Embase, Cochrane Library, and Clinicaltrials.gov for randomized controlled trials (RCTs) that were performed to evaluate TXA vs. placebo for ICH up to February 2021. The data were assessed by Review Manager 5.3 software. The risk ratio (RR) and mean difference were analyzed using dichotomous outcomes and continuous outcomes, respectively, with a fixed effect model.

**Results:** We collected 2,479 patients from four RCTs. Then, we took the change of hematoma volume, modified Rankin Scale (mRS), and adverse events as evaluation standard of the treatment for ICH. Through statistical analysis, we found that there is no obvious hematoma expansion effect after the application of TXA (RR = 1.05), and we proceeded the quantitative analysis of percentage change in hematoma volume from baseline, indicating that TXA could inhibit the expansion of hematoma volume (RR = −2.02) compared with placebo. However, according to the outcomes of mRS (0–1, RR = 1.04; 0–2, RR = 0.96), TXA cannot improve neurological functional prognosis. As for the security outcomes—mortality (RR = 1.02), thromboembolic events (RR = 0.99), neurological deterioration (RR = 0.92), infection (RR = 0.86), and craniotomy (RR = 0.41), there seems exist no statistical difference between TXA and placebo.

**Conclusions:** TXA has an advantage in the aspect of preventing hematoma expansion compared with placebo for ICH, but cannot illustrate the efficacy of TXA in improving neurological functional prognosis, which still needs more researches with large sample sizes. Moreover, for safety, we did not find obvious statistical difference between TXA and placebo.

## Introduction

Intracerebral hemorrhage (ICH) refers to spontaneous, non-traumatic bleeding occurring in the brain parenchyma, which is one of leading causes of death and disability around the world (1). Generally, it is often caused by small vessel diseases, just like hypertensive arteriopathy or cerebral amyloid angiopathy (2). As serious neurological diseases, the clinical manifestations of ICH commonly include consciousness, motor weakness, nausea, vomiting, and so on (2–4). Sadly, most of the symptoms will lead to a poor prognosis and impact the normal life of patients seriously, if they cannot receive prompt treatment (5). Moreover, with the change of fibrinolysis and coagulopathy change, ICH may expand after bleeding (6–8). So, controlling progressive factors of the hematoma and choosing the appropriate treatment to avoid the deterioration of ICH seem particularly important (9). Among the masses of factors, the change of hematoma volume highly determines the prognosis of ICH (10). Therefore, it is significant to maintain the hematoma volume, which no longer expands or even reduce. Generally, intracranial evacuation of hematoma is effective for controlling hematoma volume, while it induced surgical injury inevitably (11, 12). Maybe, medication also deserves our attention, which causes relatively little craniocerebral injury and controllable prognosis.

Tranexamic acid (TXA) is an antifibrinolytic amino acid derivative that is derived from lysine, acting as a competitive inhibitor to block the interaction between plasmin and fibrin (13, 14). During the past decade, TXA has been widely used to treat or prevent excessive blood loss (15). In addition, previous studies have found that TXA can inhibit hematoma expansion after ICH, but there is still a lack of quantitative analysis of hematoma volume changes after the application of TXA (16, 17). Moreover, the function of TXA in improving neurological functional prognosis is still controversial. So, we searched and probed randomized controlled trials (RCTs) so far to conduct a meta-analysis that aims to estimate the function of TXA in reducing hematoma volume and improving prognosis. Certainly, the safety of TXA during the treatment of ICH is also in the scope of our research (18–21), mainly considering the occurrence of adverse events like mortality, thromboembolic events, neurological deterioration, infection, and so on.

## Methods

### Study Protocol

Before we started the study, we drafted a research protocol by following the Cochrane Collaboration format (22).

### Search Strategy

Original researches in the MEDLINE, Embase, Cochrane Library, and Clinicaltrials.gov were searched using the following terms: [(“Tranexamic acid and Spontaneous intracerebral hemorrhage”) (“TXA and Spontaneous intracerebral hemorrhage”)] until February 2021. Moreover, to make sure all relevant studies have been included, we screened reference lists of relevant articles manually.

### Study Selection

Studies were included as follows: (1) study type was randomized clinical trials; (2) language restriction: only English; (3) participating patients: patients with spontaneous intracranial hematoma; and (4) intervention: TXA or placebo.

Studies were excluded as follows: (1) types of study: retrospective studies, cohort studies, reviews, and case reports; (2) control: active control; and (3) types of hemorrhage: subarachnoid hemorrhage and traumatic brain injury.

### Data Extraction

All the data were extracted independently by 2 investigators (Zeya Yan and Shujun Chen), and any disagreements were settled through discussion. After several selections and assessments, the basic information of the included trails (first author, publication, centers, and treatment groups), patient characteristics (total number, mean age, and gender), study period, and outcome events were used to extract the data (Table 1).

**Table 1:** Characteristics of the included studies and outcome events.

**Study (Clinical trial registration)**	**Centers**	**Publication**	**Treatment group, (No. of participants)**	**Total number**	**Mean age (Years)**	**Male (%)**	**Study period**	**Mainly included Outcome Events**
Sprigg et al. (19) (ISRCTN50867461)	1	Journal of Stroke and Cerebrovascular Diseases	TXA(*n* = 16)*vs*. PLA (*n* = 8)	24	TXA(67.9)*vs*. PLA(68.5)*vs*. Total(68.1)	TXA(62.5*vs*. PLA(50)*vs*. Total(62.5)	12 months	A, B, C, D, E, F, G
Arumugam et al. (18) (-)	1	The Malaysian journal of medical sciences	TXA(*n* = 15)*vs*. PLA(*n* = 15)	30	Total(52.9)	Total(60)	12 months	A, B, C, D, G
Sprigg et al. (20) (ISRCTN93732214)	124	Lancet	TXA(*n* = 1,161)*vs*. PLA(*n* = 1,164)	2325	TXA(69.1)*vs*. PLA(68.7)	TXA(55)*vs*. PLA(57)	55 months	A, B, C, D, E, F, H
Meretoja et al. (21) (NCT01702636)	13	Lancet Neurol	TXA(*n* = 50)*vs*. PLA(*n* = 50)	100	TXA(73.0)*vs*. PLA(71.0)	TXA(70)*vs*. PLA(54)	78 months	A, B, C, D, H

*PLA, Placebo; A, No hematoma expansion (the number of hematoma expansion subtract from total); B, Change in hematoma volume from baseline; C, Death; D, Thromboembolic events; E, Neurological deterioration; F, Infection; G, Craniotomy; H, mRS*.

### Outcomes

The primary outcome is hematoma volume and its change, day 1 to 2, recorded by medical imaging examination. Secondary endpoint included modified Rankin Scale (mRS) 0–1 and 0–2 at day 90. In addition, we chose the mortality, thromboembolic events, neurological deterioration, infection, and craniotomy as the safety endpoints.

### Summary Measures and Synthesis of Results

Review manager 5.3 was used to assess the data. Estimated mean differences (MDs) and estimated risk ratio (RR) (95% confidence interval [CI]) were calculated using a fixed effects model. The I^2^ statistic was used to estimate the statistical heterogeneity as follows: I^2^ < 30% represents “low heterogeneity,” 30% < I^2^ < 50% means “moderate heterogeneity,” and I^2>^ 50% means “substantial heterogeneity.” A *p*-value <0.05 was considered to be significant for all analyses, and tests are two-tailed.

### Risk of Bias

The risk-of-bias plot was assessed using Review Manager 5.3 software (The Cochrane Collaboration, Oxford, UK) for individual studies. The unified standard of the Cochrane Collaboration was applied to assess the risk of bias for RCTs, which included selection bias, performance bias, detection bias, attrition bias, reporting bias, and other potential biases.

## Results

### Search Results

**Figure 1:**
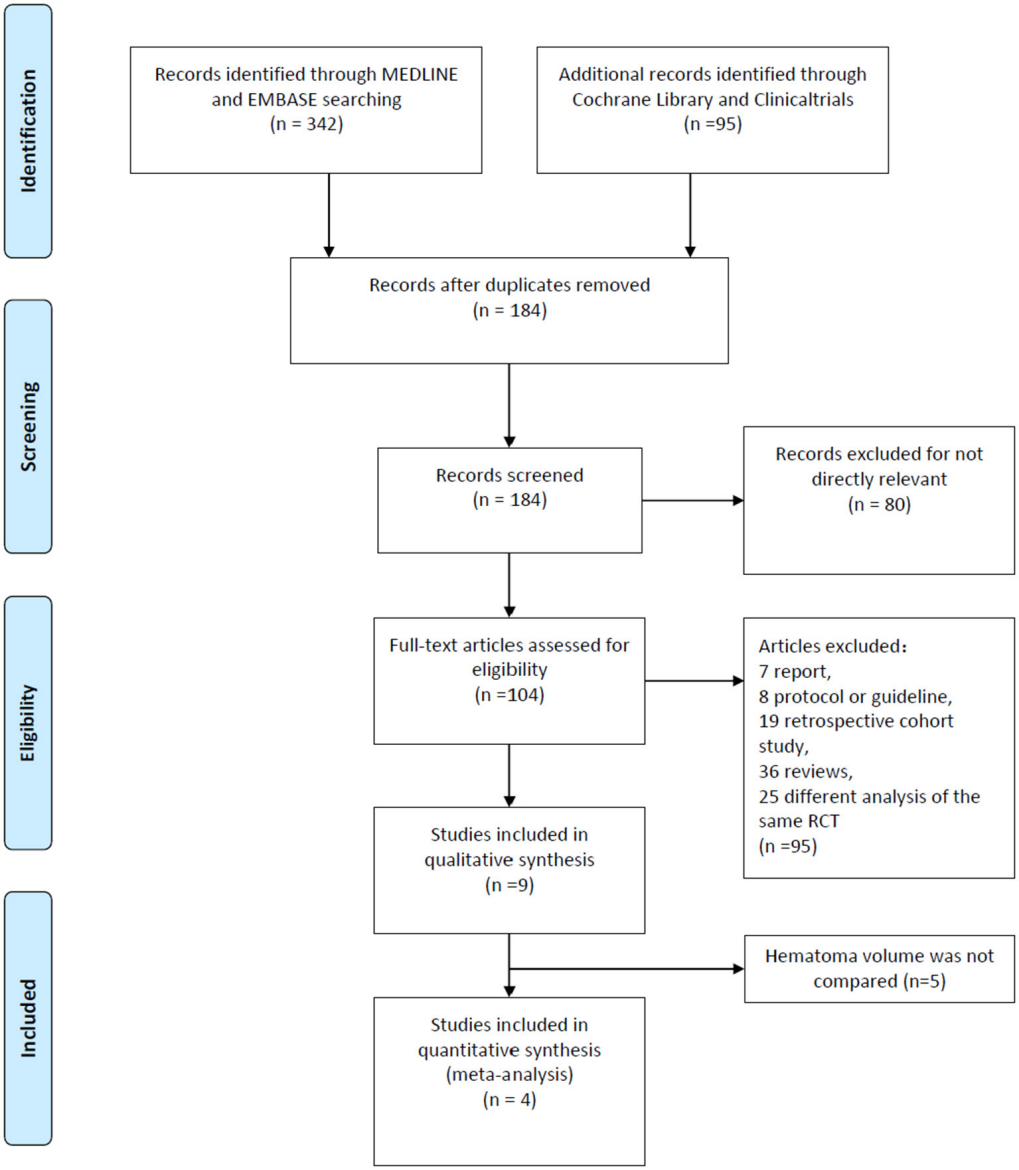
The study search, selection and inclusion process.

A total of 437 researches and abstracts from Medline, Embase, Cochrane Library, and Clinicaltrials.gov were identified. Among them, 253 studies were excluded because of duplicates. Moreover, 80 studies were excluded as they were irrelevant, such as research on other drugs or into the etiological analysis of ICH. After removing duplicates and uncorrelated titles, 104 of these articles were directly related to the topic of interest. Among them, 95 full text articles were excluded, which included 7 reports, 8 protocols or guidelines, 19 retrospective cohort studies, 36 reviews, and 25 different analyses of the same RCT. Then, we excluded the RCTs that did not contain outcomes we need. Finally, four RCTs containing 2,479 patients were included in our meta-analysis. The detailed process of screening is shown in Figure 1.

### The Change of Hematoma Volume Caused by TXA

**Figure 2:**
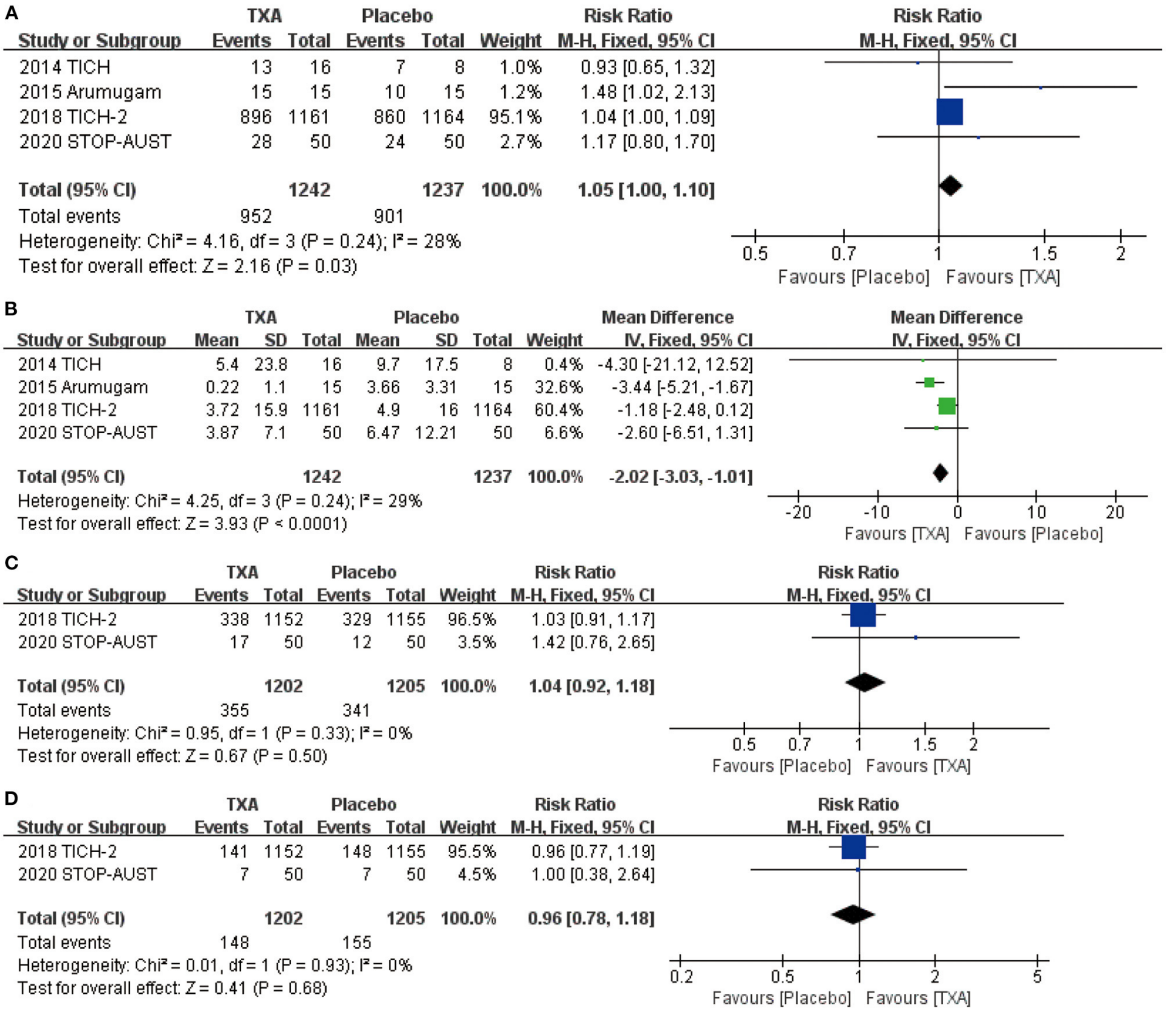
The pooled risk ratio (RR) and mean difference (MD) compared with placebo, the diamond indicates the estimated RR with 95% confidence interval (CI) for the pooled patients. **(A)** no hematoma expansion (the number of hematoma expansion subtract from total); **(B)** change in hematoma volume from baseline; **(C)** mRS (modified Rankin Scale) 0–1; **(D)** mRS (modified Rankin Scale) 0–2.

After screening and analysis of four RCTs, we firstly found there is no obvious hematoma expansion after the application of TXA compared with placebo (RR = 1.05, 95% CI: 1.00~1.10, *p* = 0.03; shown in Figure 2A). Then, we proceeded to the analysis of percentage change in hematoma volume, indicating that TXA has the effect of decreasing the volume of hematoma after the ICH (RR = −2.02, 95% CI: −3.03 to −1.01, *p* < 0.0001; shown in Figure 2B). For more details, both results above are shown in Figures 2A,B.

### Neurological Functional Prognosis After the Application of TXA

We chose the most representative outcomes at day 90 from four RCTs—mRS, to reflect the neurological functional prognosis. After the statistical analysis, both results of mRS 0–1 (RR = 1.04, 95% CI: 0.92~1.18, *p* = 0.50; shown in Figure 2C) and 0–2 (RR = 0.96, 95% CI: 0.78~1.18, *p* = 0.68; shown in Figure 2D) show no significant improvement after the application of TXA.

### Safety of the TXA for ICH

**Figure 3:**
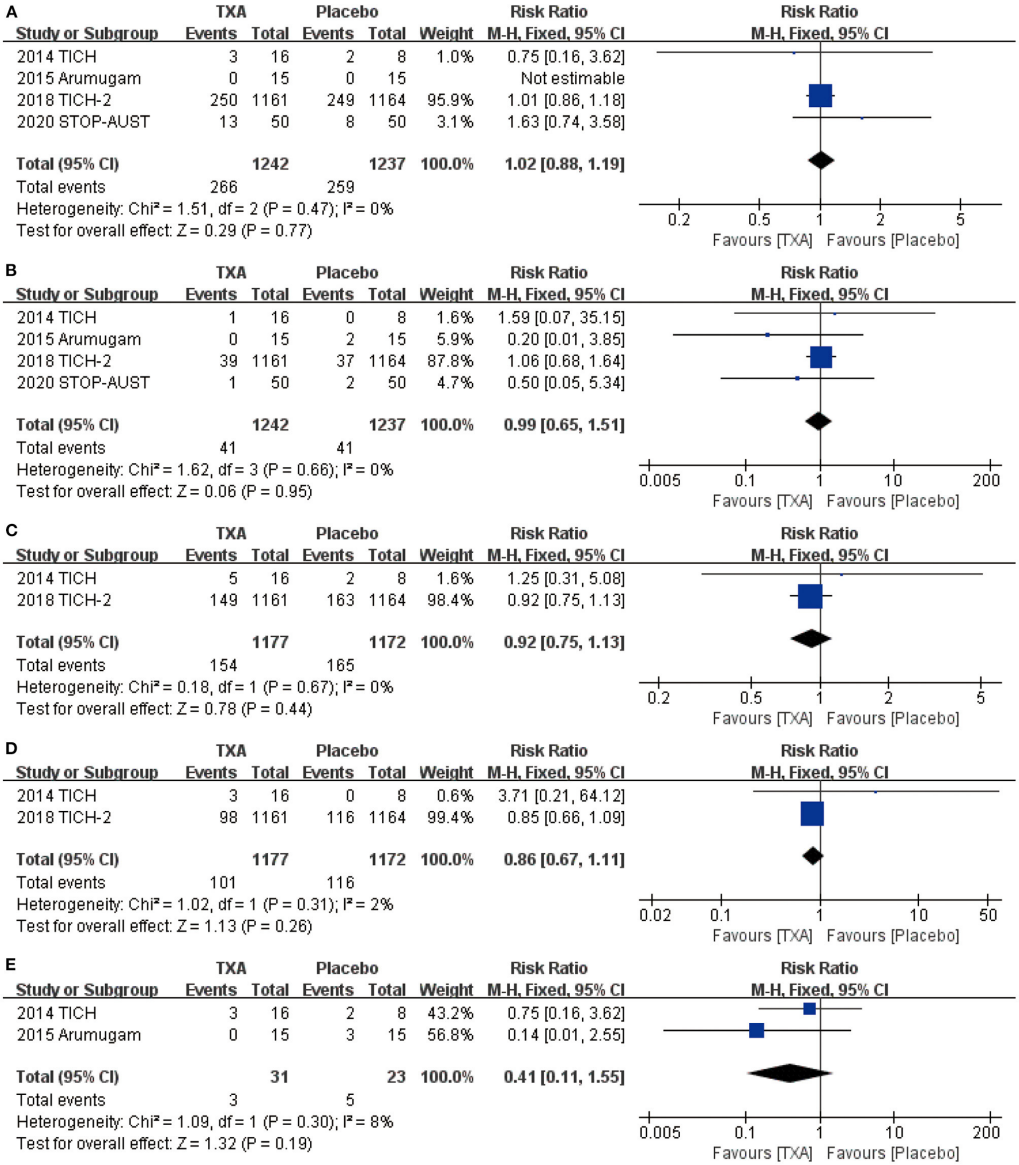
The pooled risk ratio (RR) of safety indicators compared with placebo, the diamond indicates the estimated RR with 95% confidence interval (CI) for the pooled patients. **(A)** death; **(B)**. thromboembolic events; **(C)** neurological deterioration; **(D)** infection; **(E)** craniotomy.

For the safety outcome we chose, mortality (RR = 1.02, 95% CI: 0.88~1.19, *p* = 0.77; shown in Figure 3A), thromboembolic events (RR = 0.99, 95% CI: 0.65~1.51, *p* = 0.95; shown in Figure 3B), neurological deterioration (RR = 0.92, 95% CI: 0.75~1.13, *p* = 0.44; shown in Figure 3C), infection (RR = 0.86, 95% CI: 0.67~1.11, *p* = 0.26; shown in Figure 3D), and craniotomy (RR = 0.41, 95% CI: 0.11~1.55, *p* = 0.19; shown in Figure 3E) all show no obvious statistical difference between TXA and placebo.

### Risk of Bias

**Figure 4:**
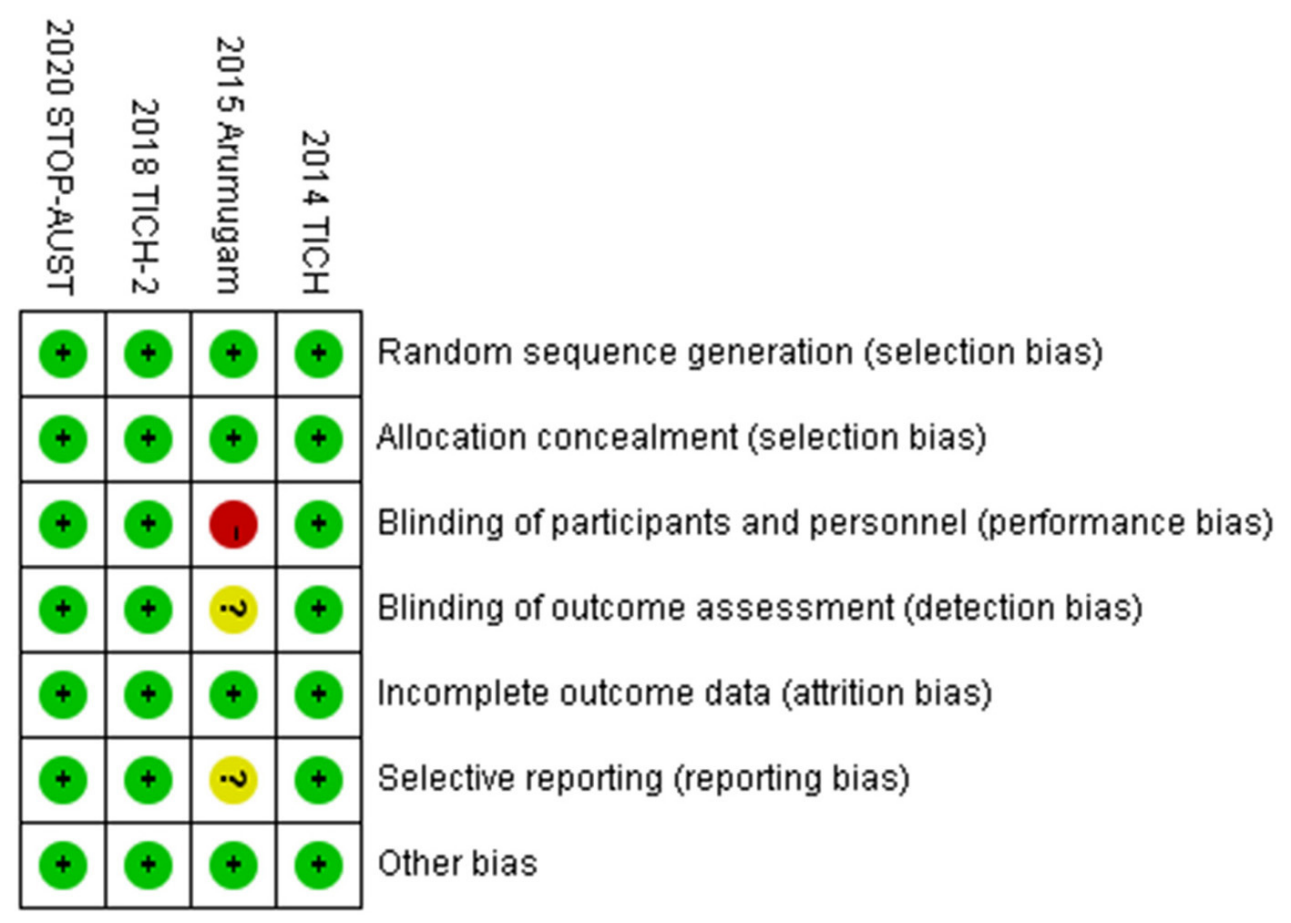
Risk of bias: a summary table for each risk of bias item for each study.

**Figure 5:**
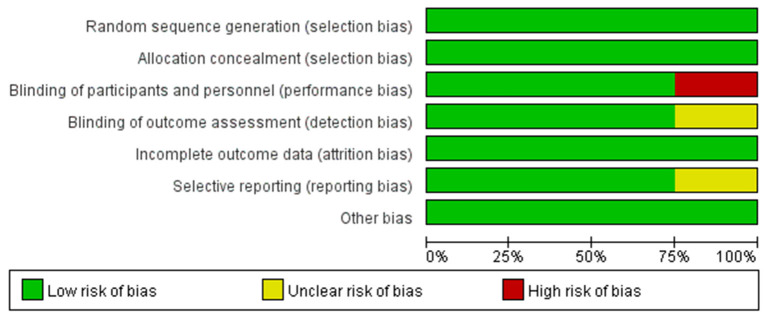
Risk of bias of all included studies.

To make the analysis of study more reliable, we analyzed the risk of bias in four RCTs (shown in Figure 4). Firstly, there was no clinical trial that had an unclear or high risk of bias in random sequence generation and allocation concealment. For blinding of outcome assessment and selective reporting, the risk of bias was clear except the study conducted by Ananda et al., while the risk of bias in this study was high for the blinding of participants and personnel. Moreover, the risk of bias in all studies was acceptable for incomplete outcome data and other bias. Generally speaking, the quality of the included RCTs in our meta-analysis was accredited (shown in Figure 5).

## Discussion

Considering the result of our study, we can conclude that TXA has an advantage in preventing hematoma expansion compared with placebo for ICH, but cannot show the efficacy in improving neurological functional prognosis. Moreover, there is no obvious statistical difference between TXA and placebo for safety.

As has been proven, TXA has the direct efficacy on reducing blood loss in many diseases (14, 23). Certainly, there exist a large amount of research exploring the mechanism or clinical function of TXA in the ICH (24, 25). They have estimated the efficacy and safety of TXA from different aspects, such as the systematic review conducted by Hu et al. (26). However, there is still no specific research to explore the function of TXA in the change of hematoma volume and neurological functional prognosis based on the statistical data from large RCTs like TICH-2 2018 (20). Therefore, we conducted this project and shared our findings through an elaborate meta-analysis.

In our study, we included four RCTs that consisted of 2,479 patients with ICH, aiming to analyze the function of TXA after ICH. During our research, we estimated the function of TXA inhibiting hematoma volume expansion and improving neurological functional prognosis. We also analyzed the safety of TXA compared with placebo after ICH.

After analysis, we found that TXA could reduce the expansion rate of hematoma volume after ICH, which is consistent with the antifibrinolytic effect of TXA, but TXA did not show obvious efficacy in improving neurological functional prognosis. In the beginning, we rearranged the data of patients whose hematoma volume was monitored by CT and conducted an analysis between the patients with hematoma expansion and those without. The result is the proportion of patients with no hematoma expansion was slightly higher in the groups that received TXA than those that received placebo. Nevertheless, this tendency is not obvious enough based on the existing research, and we also cannot ignore the influence of factors such as timing of CT scanning, different measurement of the volume, and so on. Certainly, it has revealed statistical significance and deserves more clinical research to bring the change of hematoma volume into the study indicators. Meanwhile, we can find that the majority of patients have no hematoma expansion whether they received TXA or placebo. This result is consistent with the previous study conducted by Gao et al. (27). After all, hemostasis is a spontaneous pathophysiological process, while bleeding may stop any time during the actual process of hematoma. Then, we made further analysis in terms of changes in hematoma volume from baseline to 24 h. Interestingly, the enlargement of hematoma volume in patients with hematoma expansion has direct difference between the TXA and placebo. As Figure 2B shows, TXA has the advantage of inhibiting hematoma expansion compared with placebo.

As for neurological functional prognosis, we chose mRS as the main evaluation indicator of our study. Recent research conducted by Nikola et al. and Atte et al. both made the assessment of mRS, which is the most prevalent functional outcome measure in contemporary stroke research and is recommended in international guidelines (28, 29). We included patients with mRS 0–1, firstly, with no symptoms or no disability despite symptoms, and we found there is no obvious statistical difference between the groups of TXA and placebo. So, we further relaxed the conditions and took patients with mRS 2 (slight disability but able to look after own affairs) into our study. However, the result is regrettable. The high level of mRS symbolizes the poor prognosis; so, we did not proceed to the inclusion of patients.

For the safety of TXA, whether the benefit brought by TXA is likely to be counterbalanced by harm has been controversial for a long time (30). Therefore, we analyzed the safety of TXA for the treatment of ICH as previous studies did. However, the difference is, from mass of safety indicators, we chose the numbers of patients with death, thromboembolic events, neurological deterioration, infection, and craniotomy in four RCTs to be our research objectives. In terms of these indicators, the results of the application of TXA did not exhibit obvious statistical difference compared with placebo. Certainly, it does not mean TXA is totally beneficial for the treatment of ICH.

Our meta-analysis has three main strengths. First, we concentrated on the change of hematoma volume after ICH, which has not been researched before. Second, we carried out elaborate search strategies in a variety of databases to take all appropriate RCTs into this systematic review. Third, the results of this meta-analysis can provide a better clinical choice about the use of TXA for clinicians.

Definitely, there exist few limitations in our research. Hard to ignore, the samples of TICH-2 made up the majority of patients in our meta-analysis, and it may affect the universality of our finding. Besides, for universality, the majority of patients included were from the European region like Switzerland, Turkey, and the UK. Only the study of Atte et al. contained the patients in a few medical centers from Australia and Taiwan; whether TXA could be applied to all population still lacks sufficient evidence. Moreover, four RCTs did not include all the data we need, just like mRS, which was only researched by Nikola et al. and Atte et al. The result is that we may not draw an exact conclusion. Last, the functions of TXA in preventing hematoma expansion were merely demonstrated by statistical analysis, and are still waiting for more clinical verification.

## Conclusion

To sum up, this systematic review and meta-analysis indicated TXA has an advantage in the aspect of preventing hematoma expansion compared with placebo for ICH, but cannot illustrate the efficacy of TXA in improving neurological functional prognosis, which still requires more RCTs with large sample sizes. Moreover, for safety, we did not find obvious statistical difference between TXA and placebo.

## Data Availability Statement

The original contributions presented in the study are included in the article/Supplementary Material, further inquiries can be directed to the corresponding author/s.

## Author Contributions

ZW and ZQC were the principal investigators. ZYY and SJC designed the study and developed the analysis plan. TX analyzed the data and performed the meta-analysis. XW and ZMS contributed to the writing of the article. ZQW revised the manuscript and polished the language. All authors read and approved the final submitted paper.

## Funding

This work was supported by the Suzhou Health Talents Training Project (GSWS2019002).

## Conflict of Interest

The authors declare that the research was conducted in the absence of any commercial or financial relationships that could be construed as a potential conflict of interest.

## Publisher's Note

All claims expressed in this article are solely those of the authors and do not necessarily represent those of their affiliated organizations, or those of the publisher, the editors and the reviewers. Any product that may be evaluated in this article, or claim that may be made by its manufacturer, is not guaranteed or endorsed by the publisher.
