# Supplementary material for: The Function of Tranexamic Acid to Prevent Hematoma Expansion After Intracerebral Hemorrhage: A Systematic Review and Meta-Analysis From Randomized Controlled Trials
Source: Front Neurol. 2021 Sep 24;12:710568. doi: 10.3389/fneur.2021.710568 (PMC8498595; doi:10.3389/fneur.2021.710568)
Supplement: Supplementary file 1 [file Data_Sheet_1.docx]

| Trials | TICH 2014  (ISRCTN50867461) |
| --- | --- |
| *Inclusion criteria* | Adult patients with acute (,24 hours after ictus) spontaneous ICH were identified and enrolled from the stroke service at Nottingham University Hospital NHS Trust. |
| *Exclusion*  *criteria* | The principal exclusion criteria included secondary ICH (anticoagulation, known vascular malformations), previous venous thromboembolic disease (VTE), recent (＜12 months) ischemic events (ischemic stroke [IS], myocardial infarction, peripheral artery disease [PAD]), renal impairment (estimated glomerular filtration rate, 50 mmol), and pregnancy or breast feeding. Full written informed consent was obtained from patients before randomization, or proxy consent was taken from a relative/carer if the patient lacked capacity because of being obtunded, confused, or dysphasic. |
| *Outcomes* | 1. The primary outcome was trial feasibility (surrogate for trial acceptability: number of patients screened who are eligible for enrollment and who gave informed consent);  2. Secondary outcomes included tolerability (adverse events occurring during or after administration of TA) and safety (clinical information on ischemic events [IS, transient ischemic attack, acute coronary syndrome, PAD] and VTE were also recorded). The Data Safety Monitoring Committee reviewed unblinded safety data after 6, 12, and 18 patients have been recruited and followed for 7 days;  3. Clinical measures: impairment (NIHSS) at day 7 (or discharge from hospital) and day 90 (end of follow-up); dependency (modified Rankin Scale shift), disability (Barthel Index), quality of life (EuroQoL), mood (Short Zung Depression Scale score), and cognition (telephone MMSE) at day 90;  4. Radiological measures: percentage hematoma volume change on brain imaging day 1 to day 2 and HE (defined as greater than 6-mL absolute increase in hematoma volume). All image analyses were performed blinded to clinical status and treatment allocation. CT scan data were exported from the Nottingham University Hospitals Picture Archiving and Communication System in DICOM format to an offline image analysis workstation. The data were converted to analyze format before volumetric analysis using 3DSlicer software. |
| *Conclusions* | In conclusion, we found it was feasible to administer TA in acute ICH. Larger studies, recruiting patients much earlier, are now needed to determine safety and efficacy. A number of such studies are in preparation with 2 studies currently recruiting patients. One such phase III trial, TICH-2,36 started in March 2013 and aims to recruit 2000 patients. In parallel, the STOP-AUST37 phase II trial will assess the effect of TA on hematoma expansion in“spot-positive’’ patients. |

| Trials | Arumugam 2015  (-) |
| --- | --- |
| *Inclusion criteria* | Patients aged≥18 years (either sex); Non-surgically managed patients who were evaluated by the on-call neurosurgeon and were deemed inappropriate for surgical intervention; Event within 8 hours of onset; Hypertensive intracerebral bleed; Supratentorial lesion. |
| *Exclusion*  *criteria* | Patients on anticoagulant therapy; Brainstem bleed; Intraventricular bleed on the 1st brain CT brain, including patients who developed an intraventricular bleed during the study; Malignant hypertension; Subarachnoid haemorrhage suggestive of a ruptured aneurysm; Trauma; Blood Disorder (e.g., haemophilia and idiopathic thrombocytopenic purpura); Infection (e.g., dengue haemorrhagic fever); Hepatic or renal impairment; Previous venous Recent ischaemic event (within 12 months), such as ischaemic stroke, myocardial infarction, or peripheral artery disease; and Pregnant or breast-feeding women (pregnancy was excluded in women of child-bearing age using a urine pregnancy test). |
| *Outcomes* | 1. The difference in hematoma volume (cm3) in the control group on admission and after 24 hours and between the 2nd brain computed tomography (CT) scan and the 2nd brain CT;  2. The difference in hematoma volume (cm3) in the treatment group on admission and after 24 hours and between the 1st brain computed tomography (CT) scan and the 2nd brain CT;  3. Volume expansion differences after 24 hours between the treatment and control groups. |
| *Conclusions* | This study showed a significant haematoma volume expansion in the control group compared to the treatment group. TXA is very effective in stabilising the haematoma once the patient’s blood pressure is controlled. Therefore, the present study has proven the benefits of TXA. It is important to acutely lower the systolic pressure (140 mmHg) upon admission (AHA 2010) prior to administering antifibrinolytic agents and to maintain strict blood pressure control over the following 24 hours. Therefore, every patient who presents with spontaneous ICH secondary to uncontrolled hypertension, which is nonsurgically managed, should be treated with a combination of a bolus administration of TXA (1 g) and a maintenance dose via infusion (1 g over 8 hours) with strict blood pressure control rather than blood pressure control alone. However, a multicentre double-blinded randomised study is needed to further evaluate the dose adequacy and its significance. |

| Trials | TICH-2 2018  (ISRCTN93732214) |
| --- | --- |
| *Inclusion criteria* | Adults with acute SICH within 8 h of stroke symptom onset or time last seen well. |
| *Exclusion*  *criteria* | 1. Patients with ICH secondary to anticoagulation, thrombolysis or known underlying structural abnormality such as arteriovenous malformation, aneurysm, tumor, or venous thrombosis. An underlying structural abnormality does not need to be excluded before enrolment, but where known, patients should not be recruited; 2. Contraindication to tranexamic acid. 3. Premorbid dependency (mRS > 4); 4. Concurrent participation in another drug or device trial. Participants enrolled in TICH-2 may be enrolled into the RESTART trial18 after 21 days; 5. Prestroke life expectancy < 3 months (e. g. advanced metastatic cancer). 6. Coma—Glasgow coma scale <5. 7. ICH was secondary to trauma. 8. Women of childbearing potential, pregnant, or breastfeeding at randomization. 9. Geographical or other factors that prohibit follow up at 90 days, e.g. no fixed address or telephone contact number, or overseas visitor. |
| *Outcomes* | Primary outcome: Death or dependency using the seven-level modified Rankin Scale (mRS) at day 90.  Secondary outcomes: 1. Neurological impairment (NIHSS20) at day 7 (or discharge if sooner); 2. Outcome: Disability (Barthel index21), dependency (mRS22), Quality of Life (EuroQol, EQ-5D, and EQ-VAS23), Cognition (Telephone Interview Cognition Score-Modified24), and mood (Zung Depression Scale25) at days 90 and 365. 3. Costs: Length of stay in hospital, readmission, institutionalization. 4. Radiological efficacy/safety (CT scan): Change in hematoma volume from baseline to 24 h scan, hematoma location, and new infarction. Details of hematoma volume calculation to be given in statistical analysis plan. 5. Safety endpoints recorded until day 90: Death (cause), venous thromboembolism, vascular occlusive events (stroke/transient ischemic attack/myocardial infarction/peripheral artery disease), seizures. Serious adverse events (SAEs) in first seven days. 6. MRI substudy: Prevalence of remote diffusionweighted imaging hyperintense lesions, perihematomal edema volume and diffusion restriction on day 5 MRI scan, and combined volume of the residual hematoma cavity and abnormal signal on the day 90 MRI scan. |
| *Conclusion* | In summary, tranexamic acid did not affect functional status at day 90, although potential benefits were seen with reductions in haematoma expansion, early death, and serious adverse events. The observed effect size was smaller than anticipated and is compatible with a lack of efficacy or the presence of a smaller treatment effect than expected. Future research should investigate which subgroups of patients might benefit. Tranexamic acid is inexpensive, easy to administer, seems to be safe, and is widely available, so even a modest treatment effect could have an important impact on the global scale. Larger randomised trials are warranted. |

| Trials | STOP-AUST 2020  (NCT01702636) |
| --- | --- |
| *Inclusion criteria* | Patients were eligible if they were aged 18 years or older, had a non-traumatic intracerebral haemorrhage with a spot sign, and were treatable within 4·5 h of symptom onset and within 1 h of CT angiography. Imaging for participant selection mandated a CT angiography before randomisation to demonstrate a spot sign. The spot sign was defined as contrast extravasation within the haemorrhage, assessed according to three criteria, all of which must be present: (1) serpiginous or spot-like appearance within the margin of a parenchymal haema toma without connection to an outside vessel; (2) the density (in Hounsfield units) should be greater than that of the background haematoma (site  investigators are not required to document the density); and (3) no hyper density at the corresponding location on non-contrast CT (to exclude calcium mimics). The spot signs were centrally adjudicated after database lock by two readers. |
| *Exclusion*  *criteria* | Exclusion criteria were Glascow Coma Scale score of less than 8; contraindications for antifibrinolytic therapy; very large intracerebral haemorrhage (>70 mL); brainstem haemorrhage; intracerebral haemorrhage known or suspected by study investigator to be secondary to trauma, aneurysm, vascular malformation, haemorrhagic transformation of ischaemic stroke, cerebral venous thrombosis, thrombolytic therapy, tumour, or infection; contrast already administered in 24 h before initial CT or contraindication to contrast agents; thromboembolic events in the past 12 months; planned surgery for the intracerebral haemorrhage within 24 h; hereditary or acquired haemorrhagic diathesis or coagulation factor deficiency; use of anticoagulation agents; pregnancy; concurrent use of haemostatic agents; participation in another investigational study in the past 30 days; known terminal illness; or any condition in which the study therapy is contraindicated or that could affect participation in the study, as judged by the investigator. |
| *Outcomes* | The primary outcome measure was presence of intracerebral haemorrhage growth by 24 h (±3) after start of study drug administration as defined by at least a 33% or 6mL increase from baseline, adjusted for baseline intracerebral haemorrhage volume. Secondary efficacy outcome measures were absolute intracerebral haemorrhage growth volume and absolute intraventricular haemorrhage growth volume by 24 h (±3) after start of study drug administration adjusted for baseline volumes; mRS 0–4 or return to prestroke score at 90 days; mRS 0–3 or return to prestroke score at 90 days; and categorical shift in mRS at 90 days. Secondary safety outcome measures included major thromboembolic events (myocardial infarction, ischaemic stroke, or pulmonary embolism) and death due to any cause, both by 90 days. |
| *Conclusion* | In summary, our study does not provide evidence that tranexamic acid prevents intracerebral haemorrhage growth, although the treatment was safe with no increase in thromboembolic complications. Because haemorrhage growth is a major cause of morbidity and mortality in acute intracerebral haemorrhage, new, safe, and effective treatments to stem ongoing haemorrhage are urgently required. Larger trials of tranexamic acid, with simpler recruitment methods and an earlier treatment window, are justified. |
